# Supplementary material for: A safety mechanism enables tissue-specific resistance to protein aggregation during aging in C. elegans
Source: PLoS Biol. 2023 Sep 14;21(9):e3002284. doi: 10.1371/journal.pbio.3002284 (PMC10501630; doi:10.1371/journal.pbio.3002284)
Supplement: S1 Table — (DOCX) [file pbio.3002284.s009.docx]

| Gene | Description | Human ortholog | Up-regulated by micro-sporidia [[1](#_ENREF_65)] | Differentially expressed with pharyngeal KIN-19 aggregation | Differentially expressed with pharyngeal RHO-1 aggregation | Significantly restores RHO-1 aggregation in *hsf-1(-)* |
| --- | --- | --- | --- | --- | --- | --- |
|  |  |  |  |  |  |  |
| *pals-5* | Protein containing ALS2cr12 signature |  | yes | up with *hsf-1* RNAi | up with *hsf-1* RNAi | 3/3 repeats |
| C01B10.4 | type-B carboxylesterase | Butyryl-cholinesterase  (E-value: 4e-30) |  | up with *atg-18* RNAi | up with *hsf-1* RNAi | 3/3 repeats |
| C53A5.11 | kelch domain | Actin-binding protein IPP (E-value: 1.2e-27) | yes | up with *hsf-1* RNAi | up with *hsf-1* RNAi | 3/3 repeats |
| C30H6.12 |  |  |  | up with *atg-18* RNAi | up with *hsf-1* RNAi | 3/3 repeats |
| *fbxa-30* | F-box A protein |  |  | up with *atg-18* RNAi | up with *hsf-1* RNAi | 2/3 repeats |
| F26F2.4 |  |  | yes | up with *hsf-1* RNAi | up with *hsf-1* RNAi | 2/3 repeats |
| *math-39* | MATH (meprin-associated Traf homology) domain containing |  |  | up with *hsf-1* RNAi | up with *hsf-1* RNAi | 1/3 repeats |
| T05B11.1 | F-box |  |  | up with *atg-18* RNAi | up with *hsf-1* RNAi | 1/4 repeats |
| *fbxa-86* | F-box A protein |  | yes | up with *hsf-1* RNAi | up with *hsf-1* RNAi | N.A. |
| Y105C5A.1270 |  |  |  | up with *hsf-1* RNAi | up with *hsf-1* RNAi | N.A. |
| M01G12.7 |  |  | yes | up with *hsf-1* RNAi | up with *hsf-1* RNAi | N.A. |
| K06A9.1 |  |  |  | up with *atg-18* RNAi | up with *hsf-1* RNAi | N.A. |

S1 Table. Genes upregulated in response to PQC disruption and protein aggregation in the pharynx

Reference:

1. Bakowski MA, Desjardins CA, Smelkinson MG, Dunbar TL, Lopez-Moyado IF, Rifkin SA, et al. Ubiquitin-mediated response to microsporidia and virus infection in C. elegans. PLoS Pathog. 2014;10(6):e1004200. doi: 10.1371/journal.ppat.1004200. PubMed PMID: 24945527; PubMed Central PMCID: PMCPMC4063957.
