# Supplementary figures and images for: A safety mechanism enables tissue-specific resistance to protein aggregation during aging in C. elegans
Source: PLoS Biol. 2023 Sep 14;21(9):e3002284. doi: 10.1371/journal.pbio.3002284 (PMC10501630; doi:10.1371/journal.pbio.3002284)

S2C Fig

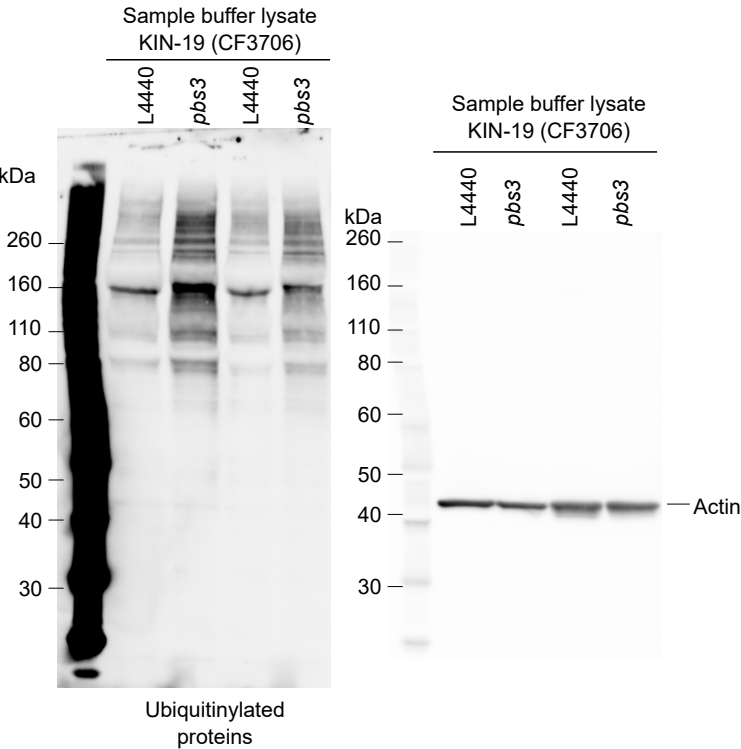

S6A Fig (Left panel)

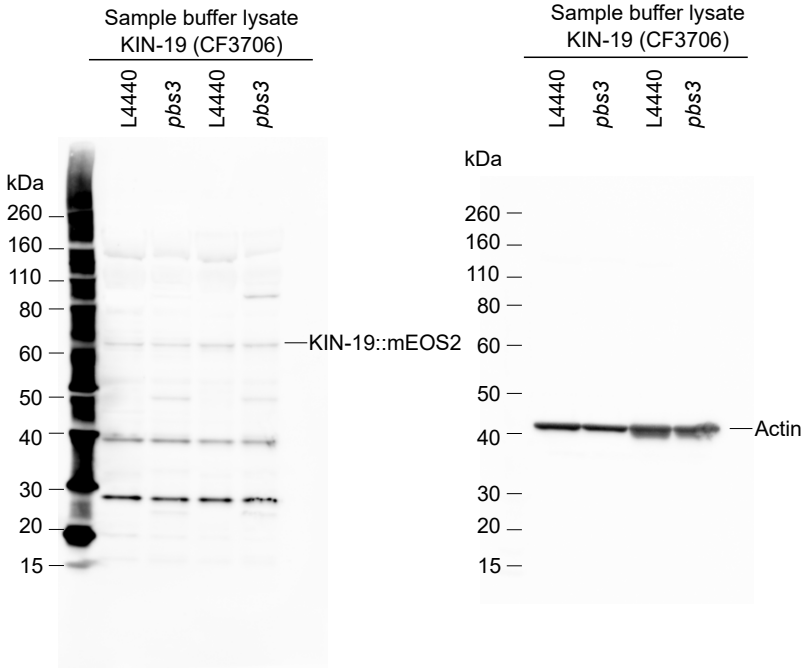

Related to S6A Fig (Left panel)

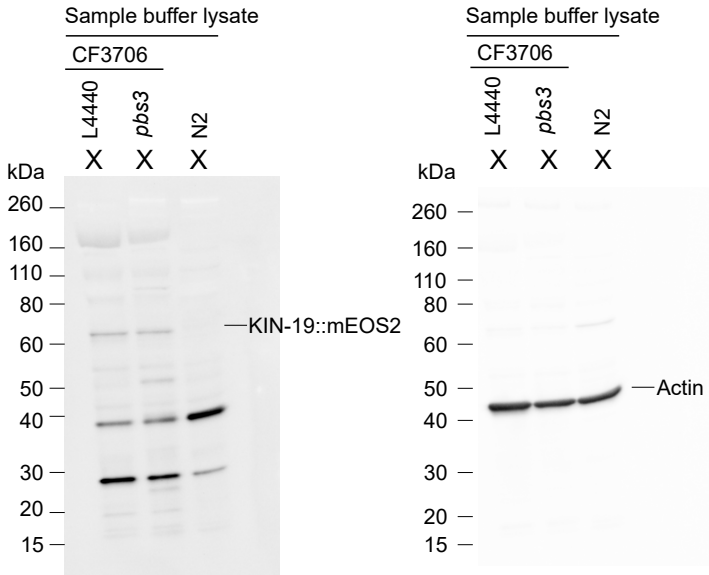

S6A Fig (Right panel)

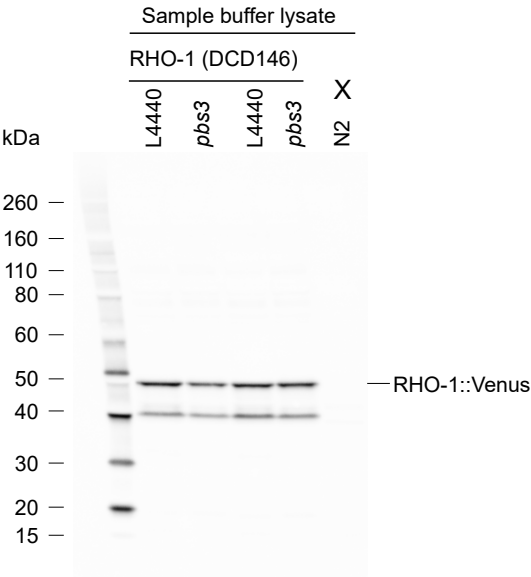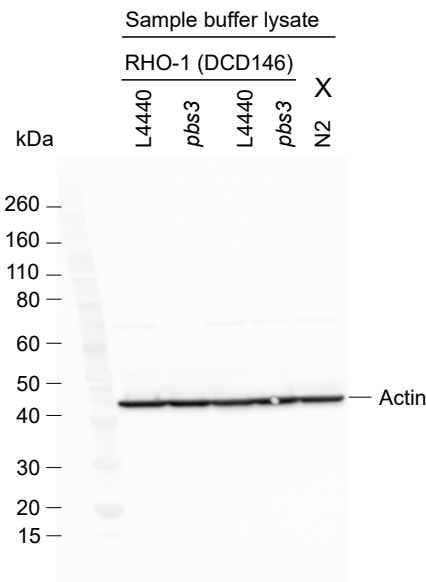

S6B Fig

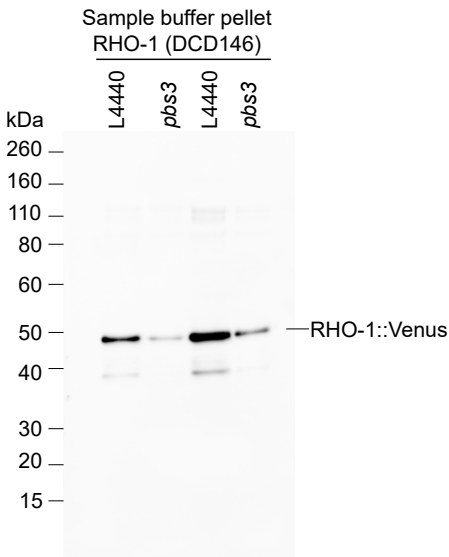

Supplement: S1 Raw Images — Blots corresponding to S2C Fig; blots corresponding to S6A Fig (Left panel); related to S6A Fig (left panel), additional blot showing band specificity of KIN-19::mEOS2 detected by anti-CK1 antibody in CF3706 transgenics and not in N2 wild-type, and corresponding anti-actin loading control blot; blots corresponding to S6A Fig (right panel); blot corresponding to S6B Fig. (PDF) [file pbio.3002284.s013.pdf]
