# Supplementary material for: A safety mechanism enables tissue-specific resistance to protein aggregation during aging in C. elegans
Source: PLoS Biol. 2023 Sep 14;21(9):e3002284. doi: 10.1371/journal.pbio.3002284 (PMC10501630; doi:10.1371/journal.pbio.3002284)
Supplement: Attachment — Submitted filename: Response to Reviewers.pdf [file pbio.3002284.s014.pdf]

We thank the reviewers for their enthusiastic remarks noting the importance of our discovery of a novel tissue-specific mechanism to prevent protein aggregation. We greatly appreciate the insightful suggestions from all reviewers on how to improve this work further. Due to my lab's move to another country and loss of group members with the necessary expertise in my group and our collaborator's group, the revisions have been greatly delayed. Nonetheless, we have performed extensive additional experiments as suggested by the reviewers to (1) characterize the protein aggregates by FLIM (new Fig. 2A), (2) reveal that impairment of a single chaperone triggers the safety mechanism (new Fig. S1E), (3) show the efficacy of RNAi in the pharyngeal muscles (new Fig. S1F), and (4) demonstrate that enhancing chaperone and proteasome activity protects against pharyngeal protein aggregation (new Fig. S2). All of these results further support our model describing a novel tissue-specific safety mechanism that prevents aggregation of newly made proteins by targeting them to the lysosome.

Reviewer #1 (Evidence, reproducibility and clarity (Required)):

Here, Jung, et al. describe a new anti-aggregation program they call SAPA that functions independently of the well-known protein quality control systems. They were addressing the question of differential tissue susceptibility to protein aggregation, and examined protein aggregation that was prevented in pharynx but not body-wall muscles when other PQC systems fail. They examined aggregation of RHO-1 in pharyngeal muscle and KIN-19 in both pharynx and body-wall muscle with age when the 3 canonical PQC systems were knocked out by RNAi of hsf-1, atg-18, and pbs-3. Surprisingly, while body-wall muscle aggregation increased, pharyngeal aggregation decreased. Next, they examined dynamics using a photoconvertible protein, and found that new aggregates appear less quickly in absence of PQC. Further experiments suggest that newly aggregated proteins are removed, rather than generally reducing translation. This process depends on lysosomal degradation, as vha-12 and scav-3 were required.
Using RNA-seq (whole body or tissue-specific is unclear from text, but logic show in S3 is explained in text), that authors identified 12 candidate genes that were upregulated in response to both pharyngeal KIN-19 and RHO-1 aggregation upon PQC failure. By testing 8 of these 12 genes by RNAi, and found that 6 contributed to SAPA. PALS-5 co-localized with aggregates and is expressed in pharynx but not body-wall muscle, and overexpression limits protein aggregation.

We thank the reviewer for pointing out the lack of clarity about the RNA-seq. We have added to this result section that the RNA-seq was done with whole animals.

My questions with the paper are largely philosophical:
1. While I agree that this phenomenon is separate from the other PQC mechanisms, is it really a truly separate phenomenon, or simply a demonstration of the intracellular pathogen response? 5 of the 12 genes from RNA-seq play a role in IPR, but does that mean that the other 7 do not, or simply haven't been tested/discovered in IPR?

We agree that the overrepresentation of genes normally upregulated in response to obligate intracellular pathogens indicates that the safety mechanism (partially or in its entirety) could be a subprogram of the intracellular pathogen response (IPR). That being said, relatively little is known about the mechanisms of the IPR, and our findings provide exciting insight. For example, in the present study, this subprogram is triggered independently of pathogens by the specific combination of local protein aggregation in the pharynx and PQC impairment. Our discovery reveals a novel function for these IPR genes, namely the

protection against endogenous protein aggregation. Until now, it has been a mystery what could be the function of most genes upregulated in the IPR and nothing is known about the five IPR genes that we identified. pals-5 and F26F2.4 are two of the most highly upregulated IPR genes and they are involved in the safety mechanism that we discovered. Bakowski et al. (10.1371/journal.ppat.1004200) showed that knock-down of either genes had no effect on pathogen load. In this current work, we demonstrate that overexpression of pals-5 is sufficient to prevent protein aggregation (Fig.5). Furthermore, the subcellular localization of PALS-5, which is the most commonly monitored IPR reporter, was not known and we have now shown its colocalization with protein aggregates. In the context of an infection, one could speculate that PALS-5 and the other IPR-related safety mechanism proteins build up tolerance to infection by improving the health of the proteome.

2. Since the assay system was designed to identify differential effects in body-wall muscle and pharyngeal muscle, what do the authors think they would have found if they had instead assayed neurons?

This is a very important question that we intend to address in the future. The diversity of proteostasis strategies between different tissues is just starting to emerge, including in neurons. Two recent collaborative studies from Dan Jarosz and Anne Brunet's labs (doi:10.1101/2022.02.26.482120 and 10.1101/2022.02.26.482115) have characterized the aggregating proteome in a wide variety of tissues in killifish, identifying a set of proteins aggregating specifically in certain tissues (independent of levels of expression). Notably, they find an enrichment of aggregating proteins with prion-like domains in the brain. Therefore, it is possible that we would find a different aggregating proteome and different proteostasis strategies in C. elegans neurons versus in pharyngeal muscles.

3. Is this system worm-specific, because of the focus on pharynx?

This is also a very important question. It is tempting to speculate that a similar safety mechanism would exist in mammals albeit using mammalian intracellular pathogen response components. There is substantial evidence linking protein aggregation and innate immune signalling. Notably, recent work from Abdel-Nour et al. (doi: 10.1126/science.aaw4144) revealed a cytoplasmic unfolded protein response using the heme-regulated inhibitor to upregulate chaperones. In turn, these chaperones work to maintain the NOD1 signalosome soluble to enable NF-kB activation. In contrast, herpesviruses promote the aggregation and subsequent degradation of signalling proteins NEMO and RIPK1 to undermine innate antiviral responses (10.1038/s41564-019-0624-1). This shows that it is important in mammalian cells to protect against endogenous protein aggregation during an intracellular pathogenic attack. The question remains whether these mechanisms are co-opted during proteostasis stress in mammalian cells as we observe in C. elegans.

4. What is the overlap of this program with genes upregulated in long-lived insulin mutants (from published data)?

We looked at the overlap between the safety program and the meta-analysis of genes annotated as upregulated in daf-2 mutants (DAF-16 targets) as previously published in Tepper et al. (10.1016/j.cell.2013.07.006). Only one gene (M01G12.7) was upregulated (p=0,036) and two of the 12 genes were not in the analysis (C30H6.12 and Y105C5A.1270). The lack of overlap is consistent with our observations that the safety mechanism is likely to be also triggered in long-lived daf-2 mutants. Our results show a further reduction of

pharyngeal KIN-19 aggregation in daf-2 mutants with pbs-3 or atg-18 RNAi treatment (one repeat, unpublished) or with hsf-1 RNAi treatment (Lechler et al. Cell Rep. 2017).

5. The use of the term "SAPA" in the abstract and results before it has been fully characterized seems premature.

We have removed the term "SAPA" from the abstract and results replacing it with the broader term "safety mechanism". Instead, we propose to call the safety mechanism "safeguard against protein aggregation" only in the discussion after it has been fully characterized in the results section. In order to emphasize that the compensatory mechanism is indeed a protective mechanism, we have changed the order and present in Fig. 2 (previously in Fig. 5) the results that preventing protein aggregation in the pharynx rescues proteotoxicity.

Reviewer #1 (Significance (Required)):

Overall, I find the experiments to be well executed and key conclusions are convincing, and it is important for the field to recognize that the previous 3 PQC systems may not be all there is, and that there are tissue-specific mechanisms of PQC.

We thank the reviewer for highlighting the quality of the experiments and importance of our discovery for the field of protein quality control.

Reviewer #2 (Evidence, reproducibility and clarity (Required)):

Summary:

The authors use C.elegans to compare two muscular tissues (pharyngeal and body wall muscle) in terms of aggregation propensity of three different proteins, KIN-19, RHO-1 and PAB-1 that have been previously studied in the David lab and how this aggregation changes upon interfering with the protein quality control machineries. The authors observe differences between the pharynx and the body wall muscle structure on how the depletion of major modulators of members of the protein quality control system affects the selected three proteins. They used photo-conversion to also study the effect of these modulators on newly synthesised protein and existing proteins. The authors claim to have identified a novel strategy termed SAPA to provide protection to the pharynx by eliminating aggregation-prone proteins in a macroautophagy-independent lysosomal manner. Yet, the presented data do not allow such bold conclusion and I will list them below in the comments.

Major comments:

1. The three major pathways of protein quality control: proteasome, macroautophagy and chaperones were studied by depleting 6 genes in total using systemic RNAi. Meaning these genes are depleted in the entire animal. There is zero discussion on trans-tissue signalling that could lead to a signalling between different tissues and an adjustment to the RNAi-mediated depletion of these pathways. How do the authors know that a RNAi-mediated depletion of e.g. pbs-3 indeed only affects the proteasome (in the studied tissues and everywhere else)? Why did the authors not express sensors for the respective pathways in the pharynx (they

already exist for body wall muscle) and first establish their RNAi-mediated approach?

We agree with the reviewer that cell non-autonomous coordination, including transcellular signalling for example involving the up-regulation of chaperones, plays an important role in proteostasis. Impairment of PQC in one tissue could lead to a compensatory upregulation of PQC in another tissue. However, our results in Fig. 4A and 4B show that inhibition of several PQC pathways together does not eliminate the safeguard response. To avoid possible adjustment to RNAi-mediated depletion of the pathways, we used available mutants to impair HSF-1 and macroautophagy throughout the animal. As impairment of the proteasome during development is lethal, we could not use null mutants for this pathway. Yet we are confident that the proteasome is effectively inhibited in the pharynx as we found a significant increase in PAB-1 aggregation in the pharynx upon proteasome inhibition by RNAi (Fig. S3). Together, these results demonstrate that reduced aggregation of RHO-1 and KIN-19 in the pharynx is not due to a compensatory upregulation of the core PQC pathways. Instead our results show that aggregation of globular proteins during PQC impairment is avoided by macroautophagy-independent targeting to the lysosome and components of the intracellular pathogen response. Still it remains possible that inter-tissue signalling is responsible for induction of this safety mechanism. Yet, a cell autonomous response is a more likely explanation because of the involvement of the intracellular pathogen response which would normally be activated in the digestive tract directly exposed to pathogen (see results with pals-5 in Fig.5 and S6). We have updated the discussion to include the possibility of cell non-autonomous regulation.

2. Neither RNAi-specificity nor effectivity were assessed in the pharynx vs. body wall muscle. What are the remaining protein or at least mRNA levels of the 6 modulators upon RNAi? Immunolabelling could enable a tissue-specific assessment of the remaining levels.

We thank the reviewer for this insightful point. We replicated our RNAi results with loss of function mutations: atg-18(gk378) mutants have a full atg-18 knockout with a deletion that removes the start codon from atg-18. unc-51(e369) is a substitution that disrupts autophagic structures (doi: 10.1080/15548627.2014.1003478). The hsf-1(sy441) mutation introduces an early stop codon resulting in a truncated HSF-1 lacking its C-terminal transactivation domain. We have added a sentence in the result section to underline our use of mutants to confirm the RNAi results.

3. It has not been addressed that the RNAi might not affect pharynx and body wall muscle alike. Why did they not employ tissue-specific depletion?

In addition to using mutants (see response to point 2), we made sure that RNAi is effective in the pharynx by controlling for GFP fluorescence levels after subjecting the animals to gfp RNAi. Using a similar experimental setup as performed for the RNAi targeting the PQC, we showed that gfp RNAi efficiently reduces GFP levels to less than 25% of control levels at day 1 of adulthood (over 4 fold reduction) (new Fig. S1F).

4. Does an over-expression of the respective gene (pbs-3, hsf-1 etc) rescue the observed effect?

We tested how enhancing PQC influences protein aggregation in the pharyngeal muscles. We found that HSF-1 overexpression or enhancing proteasome activity by RPN-6 overexpression alleviated aggregation pharyngeal protein aggregation (new Fig. S2). Thus, these core PQC

systems are normally responsible for preventing protein aggregation localized in the pharynx. These results support our conclusions that a safety mechanism is triggered because core PQC impairment renders the pharynx vulnerable to protein aggregation.

5. Not all chaperones are regulated by HSF-1. So the claim the depletion of hsf-1 could be used as tool to suppress the activity of molecular chaperones is not valid. Why not instead analyse the aggregation-propensity upon modulation of selected major chaperones such as hsp-1, daf-21 or hsp-16?

We appreciate this point, although we note that HSF-1 controls the expression of the major chaperones in basal conditions. In particular, Brunquell et al. (doi: 10.1186/s12864-016-2837-5) demonstrate that hsf-1 inhibition by RNAi leads to the downregulation of hsp-1, hsp-12, hsp-16.1, hsp-16.11, hsp-16.2, hsp-16.41, hsp-16.48, hsp-16.49 and hsp-3. Moreover, our RNA seq data (Data file S2) showed that inhibition of hsf-1 by RNAi (compared to empty vector alone) leads to the downregulation of hsp-70, hsp-110, hsp16.48, hsp-16.49, hsp-16.41 and hsp-16.2. O'Brien et al. show that hsf-1RNAi treatment reduced levels of daf-21 (10.1016/j.celrep.2018.05.093). Together these data establish that knockdown of HSF-1 is an efficient way to deplete a large selection of chaperones.
Nonetheless, following the recommendation of the reviewer, we have now evaluated the role of hsp-1 by RNAi and show that inhibiting this major chaperone leads to reduced aggregation in the pharynx (new Fig S1E). These exciting new results are consistent with our findings in hsf-1-defective animals, showing that depletion of a single chaperone is also sufficient to trigger the safety mechanism.

6. The observed reduced fluorescence levels in Fig. 1C+E upon depletion of atg-18 show an overall reduction in protein levels and not just of foci. How do the authors exclude the possibility that the overall levels of the 3 studied proteins change and not just the fraction of aggregated proteins? This is important as it is established that protein levels correlate with their aggregation-propensity.

We agree with the important point that aggregation can be affected by total protein levels. However, we can exclude an overall reduction in protein levels as inhibition of PQC (pbs-3, unc-51 and hsf-1) leads to accelerated PAB-1 aggregation in the pharynx (Fig. S3). Moreover, we can also exclude translation inhibition in response to atg-18 or pbs-3 depletion as total levels of the transcriptional reporter mEOS alone under the control of the kin-19 promoter are not reduced by these treatments (Fig. S4B).

7. I miss an analysis of the nature of the foci of the three proteins in particular upon modulation of the protein quality control pathways. The authoring lab has used in previous studies the FLIM technique. Why not here? This would add some quantitative data to this study that otherwise relies heavily on an assessment of nematodes with detectible foci. FRAP as well as biochemical fractionation would be another option.

We thank the reviewer for this helpful suggestion. We have now performed FLIM to analyse the nature of protein aggregation during PQC impairment. We show an increase in fluorescence lifetime in the pharynx of animals with PQC disruption (new Fig. 2A). This reveals that the safety mechanism triggered effectively lowers the accumulation of amyloid-like aggregates.

8. The photo-conversion approach should actually be extremely useful to identify the fate of

the photo-converted protein. If it is indeed getting degraded, a treatment with UPS or autophagy inhibitors should stabilise the protein. A chaperone-mediated disaggregation should lead to an accumulation of resolubilised soluble protein.

We thank the reviewer for highlighting the photoconversion approach to study the dynamics of protein aggregation. We followed the fate of the photoconverted red-labelled aggregates after PQC failure to investigate disaggregation. Upon inhibition of the proteasome or macroautophagy, the low rate of removal of KIN-19 aggregates was similar to control RNAi and moderately enhanced for RHO-1 aggregates (Fig. 3A to C). Therefore, disaggregation does not play a major role in the safety mechanism. In contrast, excitingly we observed a large reduction in the rate of new green-labelled aggregate formation in all conditions. As the reviewer recommends, we investigated whether soluble proteins are being degraded by measuring the total fluorescence after triggering the safety response. Indeed after 48h, we observed less total levels of newly synthesized KIN-19::mEOS while inhibiting the proteasome (pbs-3 RNAi) or inhibiting macroautophagy (atg-18 RNAi). Together, this evidence demonstrates that the KIN-19::mEOS is degraded before it assembles into aggregates.

9. I strongly recommend to analyse the protein aggregate clearance a global / proteomic and unbiased approach. An analysis of these three previously studied proteins might not be representative to draw conclusion on the protein quality control strategies for an entire tissue.

We agree that it would be desirable to perform proteomic-wide analyses. Unfortunately, it is not possible to dissect out the pharynx in C. elegans. Moreover, the techniques needed to perform tissue-specific proteomics, e.g. proximity labelling (doi: 10.1126/sciadv.1602426) or cell-selective bioorthogonal noncanonical amino acid tagging (doi: 10.1073/pnas.1421567112), have only been recently adapted to C. elegans. Therefore, we feel that the method development needed to adjust these methods to characterize tissue-specific protein aggregation is out of the scope of the current work and we plan to do this in the future.

10. On page 4 last paragraph: the authors state that they used RNAseq of "animals with pharyngeal protein aggregates but not induced PQC impairment in animals with body wall muscle aggregates" without stating the actual strains or RNAi conditions. This is not acceptable.

Extensive information related to the strains used and RNAi conditions can be found in the Materials and Methods section "Animal collection for RNA sequencing" and Figure S5 describes the analysis procedure of the RNA sequencing data. In addition, we have now included in the result section more detail related to the experimental design.

Minor comments:

1. page 3 SAPA displays aggregation-prone substrate specificity...

What is the reasoning to study PAB-1? The transition does not work and I somehow have the impression this protein was assessed simply for the reason that the reagents existed in the lab.

We thank the reviewer for highlighting this lack of clarity and we have modified this result and discussion section to explain why we choose to study PAB-1. PAB-1 differs from KIN-19

and RHO-1 because it has a low-complexity prion-like domain and aggregates after liquid-liquid phase separation into stress granules. We analysed PAB-1 because we had found previously that the mechanisms preventing PAB-1 aggregation are different from those targeting KIN-19 and RHO-1 aggregation processes (Lechler et al., Cell Rep. 2017 and Wheeler et al. bioRxiv 2019). This prior knowledge brought us to the hypothesis that the present safety mechanism could be active against globular aggregating proteins and not effective against the aggregation of stress granule proteins. The fact that the safety mechanism does not target PAB-1 also rules out a general inhibition of pharyngeal protein translation in response to PQC impairment.

2. There are some orthographical / grammatical mistakes, but nothing major.

Reviewer #2 (Significance (Required)):

This study proposes a new mechanism to protect most vulnerable tissues such as the pharynx from protein aggregation.

A number of new PQC concepts have recently been suggested, some have been substantiated by other labs, others not yet. Protein aggregates can be eliminated by cellular mechanisms such as proteolysis or disaggregation or aggregates can also be targeted to other organelles (MAGIC) or expelled (Exophers).

The target audience is the PQC / aging research community which is also my research area.

We thank the reviewer for highlighting the novelty of our findings.

Reviewer #3 (Evidence, reproducibility and clarity (Required)):

SUMMARY:

The current study focuses on the capacity of protein aggregates to hinder cellular and organismal health. Protein aggregates can occur normally, are formed in specific diseases, and are increased as an age-dependent phenomena. Altogether, these observations imply that protein maturation systems and disposal systems are known to fail (especially as a function of age) and are unable to correct specific problems in many diseases. Also, the authors note that protein aggregates are not detected in every cell of an organism and that disposal systems likely function in a cell-specific manner. Using C. elegans as a very appropriate model system, plus a plethora of techniques to substantiate their findings, the authors address their discovery of a tissue-specific system (when comparing two muscle types) that clears "aggregation-prone" proteins. Furthermore, based on their findings, and consideration of the numerous known Protein Quality Control pathways, they claim that lysosomes are utilized for this disposal process. in addition, components of the intracellular pathogen response paper to be utilized, indicating that they are co-opted.

MAJOR COMMENTS:

I have enjoyed reading the article. Moreover, I consider almost every conclusion, based on extensive analyses, to be convincing. However, I do have strong concerns about some specific statements and the interpretation of one set of experimental results.

For example:

(1) Because protein aggregation and clearance systems operate in many cellular organelles, it is important that the authors state that their studies are focused primarily on systems that operate in the cytoplasm and not the secretory pathway. In fact, this should be stated in the abstract. I make this statement because many scientists that work in the field of proteostasis actually focus on the systems that operate in a particular organelle.

We appreciate this point and have now added this to the abstract.

(2) Personally, I consider the term "aggregation-prone" to misrepresent the authors' observations. I say this because the terminology almost implies that a biological system can predict whether protein aggregation is likely. It is far more reasonable that SAPA client proteins exhibit some recognizable structure that allows/promotes selective recognition. One such structure might represent the formation of small soluble aggregates that are not recognized by their detection systems. Considering the idea that components of an intracellular pathogen response (IPR) are involved, one would be justified to tentatively argue that the IPR components might actually function as pattern recognition receptors. Of course, this is only speculative. However, this notion provides a better explanation than to indirectly imply that the proposed SAPA system can somehow recognize a protein that is likely to aggregate. Although this clarification does briefly exist in the Discussion, I strongly suggest that this notion is established at the beginning of the article and maintained throughout the text.

We thank the reviewer for pointing out this confusion. We used "aggregation-prone" as a general term to identify the proteins in the proteome that have a propensity to aggregate as determined in David et al. (PLoS Biol. 2010). At the beginning of the results section, we have updated the text to define KIN-19 and RHO-1 as "Both proteins are highly prone to aggregate with age, becoming insoluble in strong detergents and redistributing into compact puncta containing amyloid-like structures similar to disease-associated protein aggregates". Moreover, we have modified the text to avoid using "aggregation-prone" and rather used "aggregating", a term that would include the different aggregation species on the path towards the formation of large aggregates.
We agree that the biological system cannot predict aggregation and rather would recognize the unfolded newly synthesized protein or an intermediate species in the aggregation process. As the reviewer suggests, it is tempting to speculate that the IPR components recognize either the unfolded protein or intermediate aggregation species.

(3) Another major concern concerns their conclusion that lysosomes are involved. I certainly understand that knock-down of macroautophagy a gene did not exhibit an effect. However, lysosomes are known to universally require an acidic pH and membrane integrity to operate. Therefore, the inability of the loss-of-function mutations for the VHA-12 subunit and SCAV-3 do not provide supporting data. This "apparent" discrepancy must be addressed in the text. Because the current observations are not providing a detailed mechanistic understanding of how the SAPA system operates. I suspect that modification of the text will be sufficient if some alternative pathways are considered as a possibility.

We agree that the acidic environment in the lysosome and the membrane integrity of the lysosomal compartment is essential for lysosomal function. To impair lysosomal function, we

used vha-12(ok821) mutants that lack a subunit of the V-ATPase responsible for lysosomal acidification and scav-3(qx193) mutants that express a truncated version of the lysosomal membrane protein SCAV-3 required for lysosomal membrane integrity. Previous studies demonstrate that lysosome function is negatively impacted in these mutants by showing reduced levels and activity of lysosomal proteases (doi: 10.1083/jcb.201602090 and doi: 10.7554/eLife.31268). In the current study, we investigated if lysosomal activity would explain why knocking-down macroautophagy leads to less protein aggregation. Indeed, when we combined both impairment of macroautophagy (atg-18 RNAi) and impairment of lysosomes through vha-12 and scav-3 deletions, we found that levels of protein aggregation were restored to the levels observed in wild-type conditions. This double knockdown proves that lysosomes are responsible for preventing protein aggregation upon macroautophagy failure.

(4) Do do want to mention that SAPA s observed in response to total disruption of several protein quality control systems. Therefore, the study was performed under non-physiological conditions. There is nothing inherently wrong with this strategy because extreme conditions are often required to identify a phenomenon.

We agree that inhibition of the PQC by RNAi or loss-of-function mutants is likely to achieve non-physiological impairment of the PQC not normally encountered in young healthy animals. To emphasize this, we added the following: at the end of the first result paragraph "Together, these results raise the possibility that a tissue-specific mechanism is triggered in pharyngeal muscle cells to counteract vulnerability to protein aggregation caused by severe PQC inhibition", in the first paragraph of the discussion: "We describe a novel tissue-specific safety mechanism triggered in response to severe PQC impairment that limits protein aggregation and its associated proteotoxicity." and in the third discussion paragraph "We found that SAPA is triggered in response to severe perturbations of key systems maintaining protein homeostasis"

MINOR COMMENTS:

None. I consider the experimental design and statistical analyses to be appropriate.

Reviewer #3 (Significance (Required)):

As stated above, some clarifications are required to present a uniformity of thought throughout the Results section and later in the Discussion section. Nevertheless, some important questions have been addressed and answered (providing fodder for subsequent studies). I consider the use of C. elegans for this study to be appropriate. I particularly like the use of an entire organism to compare tissue-specific pathways without worry about artifacts that can be produced in dividing cells in culture. I consider the study to be significant because an explanation emerges to the question of how specific tissues can possibly respond to perturbations in the intracellular proteostasis network. Most importantly, a previously unknown system appears to be able to function in a compensatory manner. Whether this, or other, compensatory systems decline with age or are genetically defective and function as disease modifiers will be of great interest to biochemists, cell biologists, and geneticists. I

make this comment in the context of experience because I study proteostasis in mammalian cells and have generated some seminal findings.

We thank the reviewer for emphasizing the significance and novelty of the safety mechanism as tissue-specific response.

Referees cross-commenting

I consider all the reviewers' comments to be reasonable because responses by the authors will add clarity to the description of the study and the interpretation of results.
