# Supplementary material for: A safety mechanism enables tissue-specific resistance to protein aggregation during aging in C. elegans
Source: PLoS Biol. 2023 Sep 14;21(9):e3002284. doi: 10.1371/journal.pbio.3002284 (PMC10501630; doi:10.1371/journal.pbio.3002284)
Supplement: Attachment — Submitted filename: Reviews_2nd_responses.pdf [file pbio.3002284.s016.pdf]

Response to Reviewers

Note: answers by authors for this 2nd revision are highlighted in blue

Reviewers' comments

Rev. 1:
The authors have addressed my concerns.

Rev. 2:

We thank reviewer 2 for reassessing our work and we apologize for not having sufficiently addressed their concerns. In particular, it appears that our explanations have created some confusion related to the tissue-specific protection against protein aggregation and the importance of impairing distinct PQC pathways to trigger SAPA. We thank the reviewer for pointing this out and we have modified our manuscript accordingly.

Regarding the conceptual advance compared to our study published in eLife, 2019: we previously characterised age-dependent aggregates showing that they form amyloid structures similar to disease aggregates and drive functional decline. As age-dependent protein aggregation is so common, it is likely that cells have specific mechanisms to target them but we don't yet know about these mechanisms because the field has been looking at disease aggregating proteins and ectopically expressed proteins such as luciferase. Our present results reveal a completely novel mechanism using intracellular pathogen response proteins which are expressed during a proteostasis challenge to help the lysosome remove aggregating proteins despite defective macroautophagy. Another reason why we discovered this mechanism is because we are examining globular proteins intrinsically prone to aggregate (as opposed to key neurodegenerative disease related aggregating proteins, which frequently containing extensive disordered regions). In our eLife publication (and unpublished work), we made the surprising discovery that some proteins aggregate in one tissue but not another. At that timepoint, we could only speculate to why. Now we have a potential answer: a tissue-specific safeguard against protein aggregation (SAPA).

To emphasize the conceptual advance in the manuscript, we have added the following:

After "*Most of our current knowledge about the regulation of protein aggregation has been gained by examining disease-associated aggregating proteins as well as a few ectopically expressed standard aggregation-prone proteins such as luciferase*.", we added: "*Moreover, compared to aggregating proteins with disordered regions, little is known about proteostasis mechanisms specifically targeting inherently aggregation-prone globular proteins*."

In the end of the discussion, after "*In conclusion, our findings characterize a novel safety mechanism acting in a specific tissue to suppress age-dependent protein aggregation and its proteotoxicity in response to failure of core PQC systems*.", we added: "*Such mechanisms*

could be key modulators involved in defining the differences in aggregate composition between cells and tissues with age."

To address the reviewer's concerns, we have performed several additional experiments showing:

(1) Chemical inhibition of the proteasome replicates the RNAi results leading to reduced protein aggregation in the pharynx (new Fig S1E).

(2) Inhibition of *hsp-1* results in less pharyngeal KIN-19 aggregation (new Fig S1F and G).

(3) Impaired macroautophagy in the pharynx after *atg-18* RNAi measured by autophagosome /autolysosome ratios (new Fig S2B).

(4) Impaired proteasome degradation after *pbs-3* RNAi assessed by western blot analysis of ubiquitination (new Fig S2C).

(5) Similar levels of KIN-19 and RHO-1 in the pharynx upon proteasome impairment assessed by western blot (new Fig S6).

Moreover, we repeated several experiments to gather additional images by confocal microscopy to illustrate the primary data plotted in graphs (new Fig S1G, new Fig S3D and E).

Previous comment from reviewer:
Major comments:
1. The three major pathways of protein quality control: proteasome, macroautophagy and chaperones were studied by depleting 6 genes in total using systemic RNAi. Meaning these genes are depleted in the entire animal. There is zero discussion on trans-tissue signalling that could lead to a signalling between different tissues and an adjustment to the RNAi-mediated depletion of these pathways. How do the authors know that a RNAi-mediated depletion of e.g. pbs-3 indeed only affects the proteasome (in the studied tissues and everywhere else)? Why did the authors not express sensors for the respective pathways in the pharynx (they already exist for body wall muscle) and first establish their RNAi-mediated approach?

Answer by authors:
We agree with the reviewer that cell non-autonomous coordination, including transcellular signalling for example involving the up-regulation of chaperones, plays an important role in proteostasis. Impairment of PQC in one tissue could lead to a compensatory upregulation of PQC in another tissue. However, our results in Fig. 4A and 4B show that inhibition of several PQC pathways together does not eliminate the safeguard response.

Response from reviewer:
This does not address the raised point that the selected genes (for knockdown) affect one and only one specific pathway. To ensure that the model and tools used are valid, one

would need to first express reporters for the gene or function. Models for the proteasome, autophagy and protein folding in C.elegans have been established by the Holmberg, Hansen and Hartl labs, respectively. Using those models, one could then apply a knockdown by either systemically depleting the gene or better yet by using a tissue-specific knockdown approach. Next, the knockdown efficiency needs to be established (see point 2) and the effect on the selected protein quality control (PQC) pathway as well as the other remaining pathways (to ensure specificity for the PQC pathway) have to be tested.
This is important as some proteins e.g. Hsc70/HSP-1 are involved in protein folding, but also in chaperone-mediated autophagy and macroautophagy as well as targeting of protein substrates to the proteasome. So it is not as easy to dissect the different branches of the PQC and hence requiring a validation of the employed tools and models. And this does not even yet take into account the possibility of a crosstalk between the PQC pathways to adjust each other in response to an inhibition of one branch.

Answer by authors:
We understand that the reviewer asks if the knockdown of selected genes affects only one specific PQC pathway. First, it is important to note that we are using standard knockdown interventions, which have been well characterised (see detailed answer below). Second, we do not claim tissue-specificity of the knock down of PQC (see detailed answer below). Third, we make no claim that knockdown of one PQC pathway only affects that specific pathway and we completely agree with the reviewer that cross-talk will occur (see detailed answer below). **The important finding is that knockdown of core PQC pathways (directly or indirectly), all produce the *same final effect*: less aggregation of globular proteins in the pharyngeal muscles and more aggregation of globular proteins in the body-wall muscles.**

We have used well established and previously validated RNAi treatments and mutants which are commonly used in *C. elegans* to investigate proteostasis. We impaired macroautophagy by targeting both *atg-18* and *unc-51*. Both interventions have been extensively characterised e.g in the following publications: 10.1016/j.devcel.2011.06.024, 10.7554/eLife, 10.3390/cells8030236, 10.1534/genetics.107.075762. Using the protocol published by the Hansen group (10.7554/eLife) we have now added a quantification of the autophagosome /autolysosome levels in the pharynx demonstrating inefficient macroautophagy in the pharynx upon *atg-18* RNAi treatment (new Fig. S2B). We impaired chaperone expression by targeting HSF-1 both by RNAi and using the *hsf-1(sy441)* mutant. This method to reduce transcription of chaperones has been extensively characterised e.g. in the following publications (now cited in the manuscript): 10.1534/genetics.104.028423, 10.1186/s12864-016-2837-5, doi.org/10.1091/mbc.e03-07-0532. Our RNA sequencing results demonstrate reduction of chaperone levels with *hsf-1* RNAi treatment. We impaired the proteasome function by knocking down three different subunits (*pbs-3*, *rpt-6* and *pas-6*). Knocking down the proteasome by targeting the subunits by RNAi has been extensively documented in *C. elegans*, e.g. 10.7554/eLife.17721, 10.1073/pnas.0700638104, 10.1152/ajpcell.00450.2008. In response to the reviewer's request, we have replicated these results with chemical inhibition of the proteasome (new Fig. S1E) and we show accumulation of ubiquitinated proteins after *pbs-3* RNAi treatment (new Fig. S2C). In summary, considering the published literature and our additional characterizations, the

most reasonable conclusion is that the three canonical core PQC systems: chaperone-mediated folding, degradation by the proteasome and macroautophagy are effectively impaired in *C. elegans* using these interventions.

More generally, we do not claim tissue-specificity of the knock down of PQC but rather tissue-specificity of the *effects* of the knock down: The origin of the signal(s) activating SAPA could be a combination of cell autonomous and systemic PQC failure. Yet ultimately, the signals initiated by the proteostasis challenge trigger a safety mechanism acting in the pharynx to prevent aggregation and not in the body-wall muscles.
We have incorporated the following modifications to clarify this:
Title has been modified to "*Safety mechanism enables tissue-specific resistance to protein aggregation during aging*"
End of first result paragraph: "*Together, these results raise the possibility that a mechanism is triggered in pharyngeal muscle cells to counteract local vulnerability to protein aggregation, caused by severe and systemic PQC inhibition*".
Discussion, first paragraph: "*We describe a novel safety mechanism triggered to ensure tissue-specific resistance to protein aggregation and its associated proteotoxicity in response to severe and systemic PQC impairment*."
Third paragraph: "*We found that SAPA is triggered in response to severe systemic perturbations of key systems maintaining protein homeostasis*"
End of discussion: "*In conclusion, our findings characterize a novel safety mechanism acting in a specific tissue to suppress age-dependent protein aggregation and its proteotoxicity in response to failure of core PQC systems*."

We agree with the reviewer that knockdown of autophagy components, proteasome components or a reduction in chaperones by targeting HSF-1 will induce cross-talk leading to secondary effects on the other PQC pathways. As the reviewer correctly states, a compensatory upregulation of one PQC system in adjustment to the impairment of another PQC system could occur. This rationale indeed informed our decision to test combined impairment of both macroautophagy and proteasome or macroautophagy and HSF-1 (Fig. S7). The data gathered does not provide any evidence that compensatory upregulation of the three core PQC pathways would prevent protein aggregation in the pharynx. We agree with the reviewer that cross-inhibition of PQC pathways could also occur. Cross-inhibition would likely enhance the safety mechanism and resulting protection against protein aggregation in the pharynx. Notably, it is interesting that the combined knockdown of *atg-18* by RNAi in the *hsf-1(-)* mutant background significantly reduced RHO-1 aggregation compared to *atg-18* RNAi alone. This reveals potential additional benefits by disrupting chaperone expression and macroautophagy simultaneously. We feel that examining cross-inhibition is out of the scope of this current work.

Answer by authors:
To avoid possible adjustment to RNAi-mediated depletion of the pathways, we used available mutants to impair HSF-1 and macroautophagy throughout the animal. As impairment of the proteasome during development is lethal, we could not use null mutants for this pathway. Yet we are confident that the proteasome is effectively inhibited in the

pharynx as we found a significant increase in PAB-1 aggregation in the pharynx upon proteasome inhibition by RNAi (Fig. S3).

Response from reviewer:
This observation is not an evidence of the effectiveness of the RNAi of proteasomal genes. If at all, the protein levels of PAB-1 need to be tested first.

Answer by authors:
We have replicated the effects on protein aggregation in the pharynx with the RNAi of proteasomal genes by using chemical inhibition of the proteasome (new Fig S1E). Moreover, the accumulation of ubiquitinated proteins in the lysates of animals treated with *pbs-3* RNAi demonstrates the effectiveness of the proteasomal inhibition (new Fig S2C).

Answer by authors:
Together, these results demonstrate that reduced aggregation of RHO-1 and KIN-19 in the pharynx is not due to a compensatory upregulation of the core PQC pathways.

Response from reviewer:
See the comments above.

See response above.

Answer by authors:
Instead our results show that aggregation of globular proteins during PQC impairment is avoided by macroautophagy-independent targeting to the lysosome and components of the intracellular pathogen response.

Response from reviewer:
How is this supposed to work? There are excellent imaging tools available to study the lysosomal pathway. These should be used to track the degradation of RHO-1 and KIN-19 in ideally time lapse imaging analyses to support the claim that these proteins are degraded by the lysosome yet in a macroautophagy-independent manner.

Answer by authors:
Unfortunately, time lapse imaging is technically highly challenging in *C. elegans* as it requires a microfluidic device for high resolution microscopy, to repeatedly immobilize the same animal without damaging it and to avoid using standard immobilisation methods, which induce stress and impair recovery of the animals. Moreover, in order to observe the degradation of aggregation-prone KIN-19 or RHO-1 in the lysosome, we would need to inhibit lysosomal proteases without impairing lysosomal integrity. To our knowledge, no similar study has been done in *C. elegans*. Thus, we feel that this challenging request is out of the scope of this current work. By following the fate of newly synthesised KIN-19 and RHO-1 (Fig S5B), we show no accumulation of newly synthesized proteins upon macroautophagy impairment. Furthermore, our current work provides strong genetic evidence implicating macroautophagy-independent lysosomal degradation by fully rescuing

protein aggregation in animals with double knockdown of macroautophagy and lysosomal functions (Fig 4 A and B).

Answer by authors:
Still it remains possible that inter-tissue signalling is responsible for induction of this safety mechanism. Yet, a cell autonomous response is a more likely explanation because of the involvement of the intracellular pathogen response which would normally be activated in the digestive tract directly exposed to pathogen (see results with pals-5 in Fig.5 and S6). We have updated the discussion to include the possibility of cell non- autonomous regulation.

previous comment from reviewer:
2. Neither RNAi-specificity nor effectivity were assessed in the pharynx vs. body wall muscle. What are the remaining protein or at least mRNA levels of the 6 modulators upon RNAi? Immunolabelling could enable a tissue-specific assessment of the remaining levels.

Answer by authors:
We thank the reviewer for this insightful point. We replicated our RNAi results with loss of function mutations: atg-18(gk378) mutants have a full atg-18 knockout with a deletion that removes the start codon from atg-18. unc-51(e369) is a substitution that disrupts autophagic structures (doi: 10.1080/15548627.2014.1003478). The hsf-1(sy441) mutation introduces an early stop codon resulting in a truncated HSF-1 lacking its C-terminal transactivation domain. We have added a sentence in the result section to underline our use of mutants to confirm the RNAi results.

Response from reviewer:
The authors have used three mutants that affect 2 pathways (autophagy and induction of chaperones). The possibility of using inhibitors for e.g. the proteasome (MG132, epoxomicin, bortezomib) was not considered.

Answer by authors:
Following the recommendation of the reviewer, we adapted a liquid culture setup in a 96 well format to evaluate the effect of exposure to MG-132. In this new figure S1E, we replicated our RNAi results showing that chemical inhibition of the proteasome leads to less RHO-1 aggregation in the pharynx.

previous comment from reviewer:
3. It has not been addressed that the RNAi might not affect pharynx and body wall muscle alike. Why did they not employ tissue-specific depletion?

Answer by authors:
In addition to using mutants (see response to point 2), we made sure that RNAi is effective in the pharynx by controlling for GFP fluorescence levels after subjecting the animals to gfp RNAi. Using a similar experimental setup as performed for the RNAi targeting the PQC, we

showed that gfp RNAi efficiently reduces GFP levels to less than 25% of control levels at day 1 of adulthood (over 4 fold reduction) (new Fig. S1F).

Response from reviewer:
The analysis of RNAi of gfp does not address how efficient the pbs-3, hsf-1, atg-18 are depleted in the two muscle tissues.

Answer by authors:
We obtained the same results with both RNAi and mutants. Please note that RNAi is highly effective throughout in *C. elegans* due to the expression of the dsRNA transporter SID-1 expressed in all non-neuronal cells (10.1126/science.1068836). We demonstrated that RNAi is effective in the pharynx by targeting gfp. This is a validated method, first used to demonstrate RNAi by double-stranded RNA in *C. elegans* (see seminal paper by Andy Fire and Craig Mello in Nature 1998, 10.1038/35888). We also replicated the *pbs-3* RNAi results with a proteasome chemical inhibitor. As explained above, we are not claiming tissue specificity of the knockdown but tissue-specificity of the *effects* of the knock-down.

previous comment from reviewer:
4. Does an over-expression of the respective gene (pbs-3, hsf-1 etc) rescue the observed effect?

Answer by authors:
We tested how enhancing PQC influences protein aggregation in the pharyngeal muscles. We found that HSF-1 overexpression or enhancing proteasome activity by RPN-6 overexpression alleviated aggregation pharyngeal protein aggregation (new Fig. S2). Thus, these core PQC systems are normally responsible for preventing protein aggregation localized in the pharynx. These results support our conclusions that a safety mechanism is triggered because core PQC impairment renders the pharynx vulnerable to protein aggregation.

Response from reviewer:
Why are - like in almost all data sets that were added during revision - only secondary data (plotted graphs) shown?

Answer by authors:
To quantify changes in aggregation levels during ageing, we perform a microscopy-based analysis that enables us to assess a large number of animals (usually over 100 per condition) and follow changes over time in the same population (as previously published: 10.1038/s41586-020-2461-z, 10.7554/elife.43059, 10.1016/j.celrep.2016.12.033, 10.3791/56464, 10.1371/journal.pbio.1000450). For this, we visually assess the number of foci in each individual worm in a population of animals using a fluorescence dissection microscope as described in the methods section "Aggregation quantification in vivo". The raw data with the numbers of animals evaluated in the plotted graphs are found in the Data

file S1: Source data. Representative confocal images were taken to illustrate the effects in Figure 1 and Figure S1. In addition, we have now included representative confocal images of animals subjected to *hsp-1* RNAi (new Fig. S1G) and to *pbs-3* RNAi (new Fig. 3D and E). As an illustration, we have included some representative photos of animals directly visualized with the fluorescence dissection microscope (see below). Due to time constraints, it is not feasible to take photos of over a hundred worms per condition. As stated in the methods, the visual categorization of aggregate levels was done in a blind fashion for all conditions.

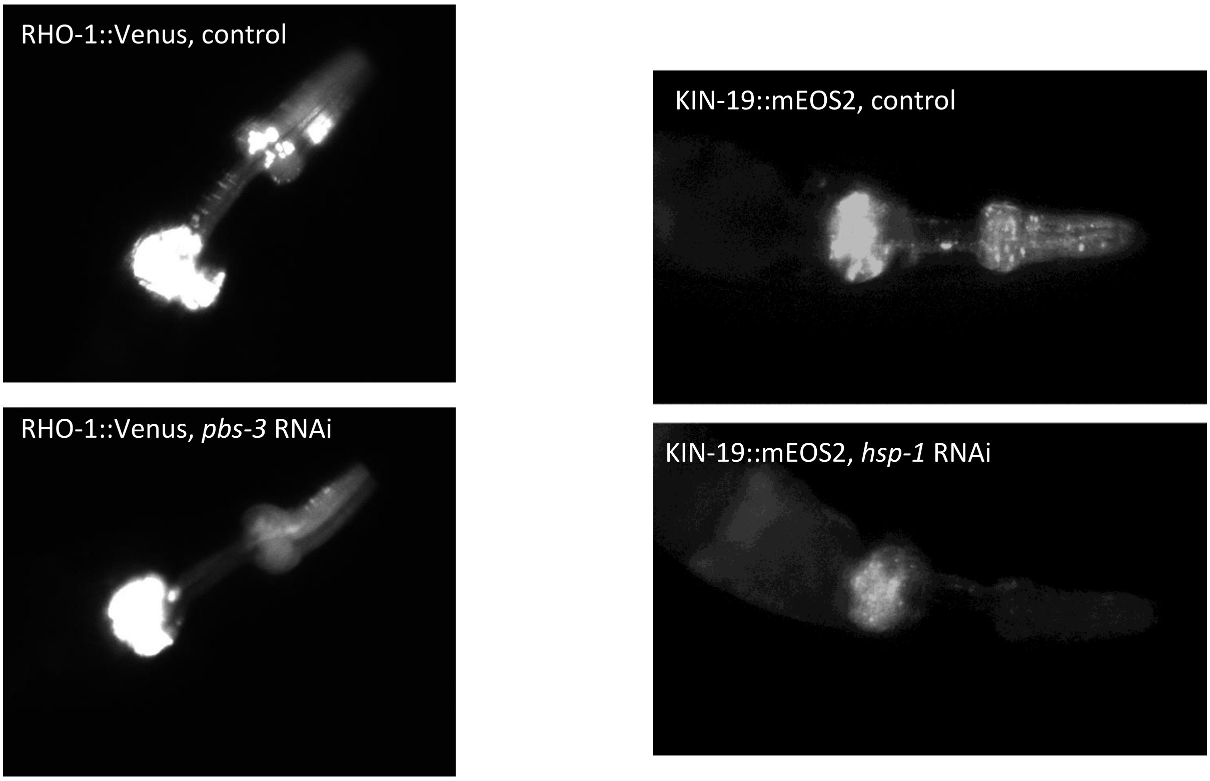

Photos taken of head regions of live animals with basic fluorescence dissection microscope. Left images show RHO-1::venus (DCD146) in pharynx at day 7. Right images show KIN-19::mEOS2 (CF3706) in pharynx at day 7.

RPN-6 and HSF-1 overexpression has been tested. An autophagy analogue is missing. One could have used a chemical activation of the autophagy pathway.
There is no demonstration that RPN-6 and HSF-1 are in fact overexpressed in the pharynx. This needs to be demonstrated.

Answer by authors:
Both models used with HSF-1 overexpression and RPN-6 overexpression have expression in the pharynx. In the corresponding model, RPN-6 is expressed under the ubiquitous *sur-5* promoter (10.1038/nature11315) and HSF-1 is expressed under its own promoter previously shown to be expressed in the pharynx (10.1111/acel.12024). We also observed that overexpression of HSF1 effectively prevents PAB-1 aggregation in the pharynx of *hsf-1(-)* mutants (10.1016/j.celrep.2016.12.033). To our knowledge, there is no genetic model available to enhance macroautophagy by overexpression of a component directly involved in macroautophagy. The standard method to enhance macroautophagy in mammalian cells

is through the chemical induced inhibition of mTOR. However, this has a variety of side effects. Moreover, it is unclear what additional information would be gained by showing that KIN-19 or RHO-1 are degraded by macroautophagy when this is artificially activated. The key finding of this work is the discovery of a novel safety mechanism triggered in response to PQC failure.

previous comment from reviewer:
5. Not all chaperones are regulated by HSF-1. So the claim the depletion of hsf-1 could be used as tool to suppress the activity of molecular chaperones is not valid. Why not instead analyse the aggregation-propensity upon modulation of selected major chaperones such as hsp-1, daf-21 or hsp-16?

Answer by authors:
We appreciate this point, although we note that HSF-1 controls the expression of the major chaperones in basal conditions. In particular, Brunquell et al. (doi: 10.1186/s12864-016-2837-5) demonstrate that hsf-1 inhibition by RNAi leads to the downregulation of hsp-1, hsp- 12, hsp-16.1, hsp-16.11, hsp-16.2, hsp-16.41, hsp-16.48, hsp-16.49 and hsp-3. Moreover, our RNA seq data (Data file S2) showed that inhibition of hsf-1 by RNAi (compared to empty vector alone) leads to the downregulation of hsp-70, hsp-110, hsp16.48, hsp-16.49, hsp-16.41 and hsp-16.2. O'Brien et al. show that hsf-1RNAi treatment reduced levels of daf-21 (10.1016/j.celrep.2018.05.093). Together these data establish that knockdown of HSF-1 is an efficient way to deplete a large selection of chaperones. Nonetheless, following the recommendation of the reviewer, we have now evaluated the role of hsp-1 by RNAi and show that inhibiting this major chaperone leads to reduced aggregation in the pharynx (new Fig S1E). These exciting new results are consistent with our findings in hsf-1-defective animals, showing that depletion of a single chaperone is also sufficient to trigger the safety mechanism.

Response from reviewer:
What is the reason that the authors only show secondary data for hsp-1 knockdown (Fig S1e). And why was only RHO-1 assessed?

Answer by authors:
We have now tested *hsp-1* RNAi on the KIN-19 model and show a similar effect (Fig. S1F). We have included confocal images of both KIN-19 and RHO-1 aggregation with the *hsp-1* treatment (new Fig. S1G). Please see explanation above related to the primary data for the knockdown of *hsp-1*.

And as above, what are the knockdown efficiencies of hsp-1 in the pharynx?

Answer by authors:
All our data imply that RNAi is effective in the *C. elegans* pharynx. First, we see similar effects with the loss of function mutants and the corresponding RNAi. Second, we showed that *gfp* RNAi is highly effective in the pharynx. Third, we show that *atg-18* RNAi effectively

causes macroautophagy disruption in the pharynx (new Fig S2B). As knockdown of *hsp-1* phenocopies the results obtained with loss of function *hsf-1* mutant, it is reasonable to conclude that *hsp-1* RNAi is effective in activating SAPA to prevent protein aggregation in the pharynx.

Were other chaperones tested as well?

Answer by authors:
Because of time constraints, we tested only HSP-1, a major chaperone in *C. elegans*, as a proof of principal that knocking down a single important chaperone activates SAPA. As we stated in our initial response, *hsf-1* knockdown is effective at decreasing a large number of chaperones in basal conditions. This demonstrates that the SAPA is triggered in response to decreased chaperone levels. It is unclear how examining further chaperone knockdowns would meaningfully improve this study.

previous comment from reviewer:
6. The observed reduced fluorescence levels in Fig. 1C+E upon depletion of atg-18 show an overall reduction in protein levels and not just of foci. How do the authors exclude the possibility that the overall levels of the 3 studied proteins change and not just the fraction of aggregated proteins? This is important as it is established that protein levels correlate with their aggregation-propensity.

Answer by authors:
We agree with the important point that aggregation can be affected by total protein levels. However, we can exclude an overall reduction in protein levels as inhibition of PQC (pbs-3, unc-51 and hsf-1) leads to accelerated PAB-1 aggregation in the pharynx (Fig. S3).

Response from reviewer:
This does not address the concern. What is needed is a quantification of the protein levels. The fluorescence levels already indicate a reduction in the protein abundance. This should be confirmed by e.g. immunostaining.

Answer by authors:
We performed western blot analysis of KIN-19::mEOS and RHO-1::tagRFP levels in the pharynx (new Fig. S6). Quantification of total lysates dissolved in loading buffer showed no clear change in levels upon proteasome knockdown (new Fig. S6A). In contrast, we found a notable reduction in the highly aggregated protein fraction in the loading buffer insoluble pellet (new Fig. S6B). The later finding correlates with the reduced level of aggregates assessed by microscopy upon proteasome knockdown. Overall, the western blot results show that the aggregating fraction is modulated in response to PQC failure rather than the overall protein levels.

Answer by authors:
Moreover, we can also exclude translation inhibition in response to atg-18 or pbs-3

depletion as total levels of the transcriptional reporter mEOS alone under the control of the kin-19 promoter are not reduced by these treatments (Fig. S4B).

Response from reviewer:
This response does not address the concern either.

We misunderstood that the reviewer was concerned that translation inhibition could be the cause of reduced protein aggregation.

previous comment from reviewer:
7. I miss an analysis of the nature of the foci of the three proteins in particular upon modulation of the protein quality control pathways. The authoring lab has used in previous studies the FLIM technique. Why not here? This would add some quantitative data to this study that otherwise relies heavily on an assessment of nematodes with detectible foci. FRAP as well as biochemical fractionation would be another option.

Answer by authors:
We thank the reviewer for this helpful suggestion. We have now performed FLIM to analyse the nature of protein aggregation during PQC impairment. We show an increase in fluorescence lifetime in the pharynx of animals with PQC disruption (new Fig. 2A). This reveals that the safety mechanism triggered effectively lowers the accumulation of amyloid-like aggregates.

Response from reviewer:
I appreciate that the authors used the FLIM technique. The reason that I asked for either FRAP or FLIM was to validate the foci as aggregated species. A much higher resolution and magnification is needed to differentiate between foci and diffuse proteins to then assess them by FLIM. Alternatively, use FRAP to analyze the mobility of the foci.

Answer by authors:
We have extensively validated the foci as aggregated species by FRAP in our previous publications. Please see Figure 2, Table 1, Figure 6 (10.1371/journal.pbio.1000450), Figure 6B (10.1016/j.celrep.2016.12.033), Figure 1 - figure supplement 1A-D and Figure 2 – figure supplement 2C-E (10.7554/eLife.43059.006). In the present study, we found that the number of foci is reduced in the pharynx during PQC impairment and this was independently confirmed by FLIM showing lower levels of amyloid-like aggregates. Please note that FLIM is not a high-resolution technique. We have updated the manuscript to clarify that the models have been previously validated by FRAP: "*Both globular proteins are highly prone to aggregate with age, becoming insoluble in strong detergents and redistributing into compact immobile puncta as assessed by the absence of fluorescence recovery after photobleaching*.".

previous comment from reviewer:
8. The photo-conversion approach should actually be extremely useful to identify the fate of

the photo-converted protein. If it is indeed getting degraded, a treatment with UPS or autophagy inhibitors should stabilise the protein. A chaperone-mediated disaggregation should lead to an accumulation of resolubilised soluble protein.

Answer by authors:
We thank the reviewer for highlighting the photoconversion approach to study the dynamics of protein aggregation. We followed the fate of the photoconverted red-labelled aggregates after PQC failure to investigate disaggregation. Upon inhibition of the proteasome or macroautophagy, the low rate of removal of KIN-19 aggregates was similar to control RNAi and moderately enhanced for RHO-1 aggregates (Fig. 3A to C). Therefore, disaggregation does not play a major role in the safety mechanism. In contrast, excitingly we observed a large reduction in the rate of new green-labelled aggregate formation in all conditions. As the reviewer recommends, we investigated whether soluble proteins are being degraded by measuring the total fluorescence after triggering the safety response. Indeed after 48h, we observed less total levels of newly synthesized KIN-19::mEOS while inhibiting the proteasome (pbs-3 RNAi) or inhibiting macroautophagy (atg-18 RNAi). Together, this evidence demonstrates that the KIN-19::mEOS is degraded before it assembles into aggregates.

Response from reviewer:
Such an analysis (ideally depicted in a time lapse manner) would allow to track individual foci to literally see if they get degraded or resolubilized in the different genetic backgrounds. The depicted plotted graphs (no imaging raw data) do not allow an assessment.

Answer by authors:
As discussed above, a microfluidic system would be needed to repeatedly immobilise the animals without damaging them for a time lapse tracking of individual foci. Yet we can address this at the population level. Indeed, we are assessing the aggregate levels over time in the same population of worms (shown in the depicted plotted graphs in Figure 3A to C). If the foci were significantly degraded or resolubilized in response to PQC failure, we would observe an increase with time (at 24h and 48h after the photoconversion) in the number of animals with low levels of photoconverted red emitting foci compared to control conditions, as discussed above. The most striking difference seen in the representative confocal images for KIN-19::mEOS (new Fig. 3D) and RHO-1::mEOS (new Fig. 3E) taken 48h after conversion is the relative absence of newly synthesised protein (labelled in green) coating the old foci (labelled in magenta) or the formation of new foci in the *pbs-3* RNAi treated animals. We have updated this section in the results to clarify our findings.

previous comment from reviewer:
9. I strongly recommend to analyse the protein aggregate clearance a global / proteomic and unbiased approach. An analysis of these three previously studied proteins might not be representative to draw conclusion on the protein quality control strategies for an entire tissue.

Answer by authors:
We agree that it would be desirable to perform proteomic-wide analyses. Unfortunately, it is not possible to dissect out the pharynx in C. elegans. Moreover, the techniques needed to perform tissue-specific proteomics, e.g. proximity labelling (doi: 10.1126/sciadv.1602426) or cell-selective bioorthogonal noncanonical amino acid tagging (doi: 10.1073/pnas.1421567112), have only been recently adapted to C. elegans. Therefore, we feel that the method development needed to adjust these methods to characterize tissue-specific protein aggregation is out of the scope of the current work and we plan to do this in the future.

Response from reviewer:
If an unbiased approach is not possible, the authors should avoid generalizing claims and only refer to RHO-1, KIN-19 and PAB-1.

Answer by authors:
We have modified the following section titles to emphasize the focus on KIN-19 and RHO-1.

"Aggregation of KIN-19 and RHO-1 is reduced in pharyngeal muscles in response to PQC impairment"

"The safety mechanism prevents de novo formation of KIN-19 and RHO-1 aggregates"

"Macroautophagy-independent lysosomal degradation protects against RHO-1 aggregation"

In the introduction:
"This safety mechanism is triggered in response to defective conventional PQC and it is highly effective at preventing the aggregation of globular proteins, KIN-19 and RHO-1."

previous comment from reviewer:
10. On page 4 last paragraph: the authors state that they used RNAseq of "animals with pharyngeal protein aggregates but not induced PQC impairment in animals with body wall muscle aggregates" without stating the actual strains or RNAi conditions. This is not acceptable.

Answer by authors:
Extensive information related to the strains used and RNAi conditions can be found in the Materials and Methods section "Animal collection for RNA sequencing" and Figure S5 describes the analysis procedure of the RNA sequencing data. In addition, we have now included in the result section more detail related to the experimental design.

Response from reviewer:
Ok.

Rev. 3: Richard Sifers – note that this reviewer has signed his review
The authors successfully responded to my original comments. Their conclusions are now intact because they are supported by additional experimentation and more accurate

explanation of their findings. In my opinion, the revised manuscript describes an interesting and important study worthy of publication in PLOS Biology.

Comments from the academic editor:

s/he has also argued that day 7 worms are not considered old, thus we would also like you to insert 'mid-life' in several places in the text to refer to these worms and acknowledge that point.

Answer by authors:
We apologize for the confusion over the word "aged" which was intended to refer to worms experiencing the aging process rather than old aged worms. We agree that Day 7 animals are considered mid-life animals. At this stage the animals are post-reproductive (doi: 10.1534/g3.113.008664). They are "aged" in the sense that effects related to the aging process such as functional decline (e.g. motility) and accumulation of protein aggregates are detected. We have modified "aged" as following:

In contrast, PQC inhibition led to a significant reduction in RHO-1 and KIN-19 aggregation in the pharyngeal muscles in both young animals (2 to 4 days of adulthood) and during the course of aging in post-reproductive animals (6 to 10 of adulthood) (Fig. 1 C to F, Fig. S1 A to D and Data File S1).

Fig. 1: *(C, D) Animals during the course of aging with impaired PQC have less KIN-19::mEOS2 aggregates in the pharyngeal muscles.*

Fig. S1. *(A, B) Animals between day 8 and day 10 of adulthood with impaired PQC have less KIN-19 aggregates in the pharynx.*

Strikingly, SAPA was so effective that animals during the mid-life aging process were protected against protein aggregation even compared to animals of similar ages, not exposed to PQC failure.

Please also note in the text that the pharynx muscles start to fail well before worms are considered "old", thus the tissues considered could age at different times.

Answer by authors:
At the beginning of the result section, we have added the following: *"Well before old age, C. elegans experiences functional decline in both tissues, potentially at different rates"*
